# Adsorption of Polyelectrolyte onto Nanosilica Synthesized from Rice Husk: Characteristics, Mechanisms, and Application for Antibiotic Removal

**DOI:** 10.3390/polym10020220

**Published:** 2018-02-23

**Authors:** Tien Duc Pham, Thu Thuy Bui, Van Thanh Nguyen, Thi Kieu Van Bui, Thi Thuy Tran, Quynh Chi Phan, Tien Dat Pham, Thu Ha Hoang

**Affiliations:** 1 Faculty of Chemistry, VNU-University of Science, Vietnam National University, Hanoi, 19 Le Thanh Tong, Hoan Kiem, Hanoi 10000, Vietnam; buithuthuy190397@gmail.com (T.T.B.); nguyenvanthanhb_t59@hus.edu.vn (V.T.N.); kieuvansp2@gmail.com (T.K.V.B.); phuongthuy.hus@gmail.com (T.T.T.); 2 HUS High School for Gifted Students, Hanoi University of Science, Vietnam National University, Hanoi, 182 Luong The Vinh, Thanh Xuan, Hanoi 10000, Vietnam; quynhchi.phan110@gmail.com; 3 High School of Education Sciences, University of Education, Vietnam National University, Hanoi, Kieu Mai, Phuc Dien, Bac Tu Liem, Hanoi 10000, Vietnam; tiendat151002@gmail.com

**Keywords:** polyelectrolyte, PDADMAC adsorption, nanosilica, rice husk, amoxicillin

## Abstract

Adsorption of the polyelectrolyte polydiallyldimethylammonium chloride (PDADMAC) onto nanosilica (SiO_2_) fabricated from rice husk was studied in this work. Nanosilica was characterized by X-ray diffraction, Fourier-transform infrared spectroscopy (FTIR), and scanning electron microscopy (SEM). Adsorption of PDADMAC onto SiO_2_ increased with increasing pH because the negative charge of SiO_2_ is higher at high pH. Adsorption isotherms of PDADMAC onto silica at different KCl concentrations were fitted well by a two-step adsorption model. Adsorption mechanisms of PDADMAC onto SiO_2_ are discussed on the basis of surface charge change, evaluation by ζ potential, surface modification by FTIR measurements, and the adsorption isotherm. The application of PDADMAC adsorption onto SiO_2_ to remove amoxicillin antibiotic (AMX) was also studied. Experimental conditions such as contact time, pH, and adsorbent dosage for removal of AMX using SiO_2_ modified with PDADMAC were systematically optimized and found to be 180 min, pH 10, and 10 mg/mL, respectively. The removal efficiency of AMX using PDADMAC-modified SiO_2_ increased significantly from 19.1% to 92.3% under optimum adsorptive conditions. We indicate that PDADMAC-modified SiO_2_ rice husk is a novel adsorbent for removal of antibiotics from aqueous solution.

## 1. Introduction

Removing organic pollutants is of great importance in environmental remediation, because numerous organic wastes are toxic. Techniques based on biological processes, chemical processes, physical processes, or a combination of these can be used to remove organic pollutants [1,2,3,4]. Processes that have been used to remove pollutants from wastewater include membrane process, ozonation [5], Fenton oxidation, chlorination, photocatalytic degradation using UV–vis radiation [6,7,8], and adsorption [9,10,11,12,13]. Some new water treatment processes for sustainability have been reported. Osmosis-membrane distillation has been used to recover water from oily wastewater, while various adsorbents have been selected to purify water. Some novel adsorbents are biochar [14], vegetable coconut palm [15], imprinted materials [16], natural soils [17,18,19], and metal oxides [20]. Recently, many studies have reported that adsorptive removal of organic contaminants from aqueous solutions is significantly enhanced by using a solid surface modified with an ionic polymer (polyelectrolyte) [21,22,23,24]. In these systems, an understanding of adsorption characteristics and mechanisms is important to control and increase the efficiency of removing organic contaminants. Therefore, adsorption of polyelectrolytes on a solid surface has been a topic of much research [25,26,27]. 

Antibiotics in the water supply is a type of organic pollutant, because antibiotic residue can cause serious problems [19,28]. Thus, removal of antibiotics by a suitable method, such as adsorption, using inexpensive adsorbents becomes preferable [19,29]. Numerous effective adsorbents for antibiotic removal have been studied [28,30,31]. Rice husk is a very popular agricultural resource in many countries. Therefore, adsorbent synthesized from rice husk is very cheap. Silica is a common adsorbent that can be easily synthesized from rice husk [32]. However, nanosilica fabricated from rice husk is a nonporous and low-charge-density material, so it is difficult to remove organic pollutants directly. In this case, surface modification of silica is necessary to enhance removal efficiency. Polydiallyldimethylammoniym chloride (PDADMAC) is a strong polycation so that its structure is independent of pH. PDADMAC can be used to modify a solid surface or to form composites, which are novel materials for removing some organic pollutants [23,33,34]. Adsorption of PDADMAC onto silica (quartz crystal) at different ionic strengths has been systematically studied [35]. However, PDADMAC adsorption onto nanosilica used for removal of beta lactam antibiotics has not been studied.

Adsorption is normally studied under isothermal conditions. For adsorption isotherms, Langmuir [36], Freundlich [37], and Brunauer–Emmett–Teller [38] models are often discussed. The Langmuir model is used to describe isotherms of some polymers at solid surfaces. However, these three models cannot be applied to polyelectrolyte adsorption. The two-step model proposed by Zhu et al. [39], with a general adsorption isotherm equation, was successfully applied to various types of adsorbates, including polyelectrolyte, for numerous systems [39,40,41]. Additionally, two-step kinetics could evaluate the growth of polyelectrolyte layers [42]. Thus, the two-step model can be used to fit adsorption isotherms of PDADMAC onto nanosilica. 

The aim of the present study is to investigate adsorption of PDADMAC onto nanosilica synthesized from rice husk. The characterizations of silica were examined by X-ray diffraction (XRD), Fourier-transform infrared spectroscopy (FTIR), scanning electron microscopy (SEM), and ζ potential measurements. The adsorption isotherm of PDADMAC on nanosilica was studied at different pH values and ionic strengths. Adsorption mechanisms are suggested based on the adsorption isotherm, surface modification by FTIR, and the change in surface charge by electrokinetic (ζ potential) measurements. The application of PDADMAC adsorption to amoxicillin antibiotic removal is also investigated. To the best of our knowledge, this is the first systematic study of PDADMAC adsorption onto nanosilica synthesized from rice husk to relate electrokinetics with FTIR spectroscopy, modeling an adsorption isotherm by the two-step model and applying it to the removal of antibiotics using PDADMAC-modified nanosilica.

## 2. Experimental 

### 2.1. Materials 

**Figure 1:**
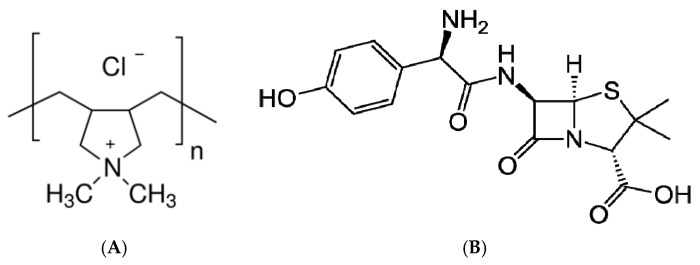
Chemical structure of polydiallyldimethylammonium chloride (PDADMAC) (**A**) amoxicillin (AMX) (**B**).

The rice husk used in the present study was collected from the Food Commerce Company (Bacninh, Vietnam). Nanosilica was synthesized by the hydrothermal method from the rice husk. Polycation polydiallyldimethylammonium chloride (Figure 1A) 20 wt % in H_2_O with a molecular weight of 400–500 kg/mol was purchased from Sigma-Aldritch. Amoxicillin trihydrate (AMX) with purity higher than 98% was supplied by Tokyo Chemical Industry (Chuo-ku, Tokyo, Japan). The chemical structure of AMX is indicated in Figure 1B. Ionic strength and pH were adjusted by the addition of KCl (p.A, Merck, Frankfurter, Germany), HCl and KOH (volumetric analysis grade, Merck). Solution pH was measured using an HI 2215 pH meter (Hanna, Woonsocket, RI, USA). The pH electrode was calibrated with three standard buffers of 4.01, 7.01, and 10.01 (Hanna). Other chemicals with analytical grade were purchased from Merck. An ultrapure water system (Labconco, Kansas City, MO, USA) with a resistivity of 18.2 MΩ was used to produce ultrapure water in preparing all aqueous solutions.

### 2.2. Fabrication of Nanosilica from Rice Husk

**Figure 2:**
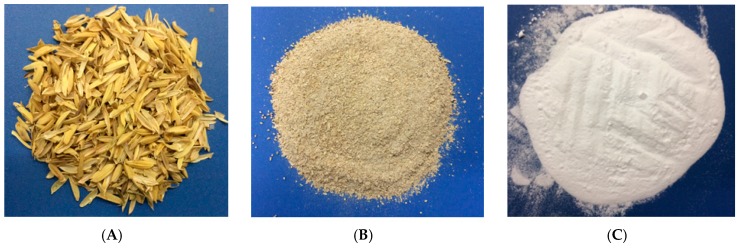
Pictures of (**A**) rice husk; (**B**) milled rice husk; and (**C**) synthesized nanosilica.

Nanosilica was synthesized from rice husk according to our previously published paper, with a minor modification [41]. About 80 g of milled rice husk was placed in a 1 L beaker containing 386 g of ultrapure water (about 5 mL per gram of milled rice husk) and 14 g of concentrated H_2_SO_4_ and stirred for at least 5 h at 120 °C. Subsequently, the pretreated milled rice husk was washed with ultrapure water until neutral pH was reached. Then, the rice husk was dried at 110 °C for 1 h and calcined in a thermal furnace at 800 °C for 12 h to obtain nanosilica [32]. After that, nanosilica was washed two times with diluted sulfuric acid (0.2 M H_2_SO_4_) before being rinsed with ultrapure water. The volumes of ultrapure water and 0.2 M H_2_SO_4_ are about 10 mL per gram of adsorbent. Finally, nanosilica particles were heated at 800 °C for 24 h and then cooled to room temperature before being dispersed in ultrapure water to form a nanosilica solution [43]. Figure 2 shows pictures of rice husk, milled rice husk, and synthesized nanosilica.

### 2.3. Characterization Methods

The nanosilica synthesized from rice husk was characterized by XRD, FTIR, SEM, and ζ potential measurements.

The XRD pattern was collected on a Bruker D8 Advance X-ray Diffractometer with CuK*_α_* radiation (*λ* = 1.5418 Å). Intensity of the diffraction peaks was recorded in the 1–70° (2θ) range with a step size of 0.03°.

The FTIR spectra were collected with an Affinity-1S spectrometer (Shimadzu, Kyoto, Japan). The FTIR spectra of PDADMAC solution, nanosilica particles, nanosilica after PDADMAC adsorption at the maximum level, and PDADMAC solution salt were obtained at 25 °C and atmospheric pressure (with nanosilica) and in PDADMAC solution at a resolution of 4 cm^−1^.

The morphology and average size of synthesized SiO_2_ were examined by SEM (Hitachi S4800, Chiyoda-ku, Tokyo, Japan). The average size of nanosilica particles was calculated by using ImageJ software.

The charging behavior of synthesized SiO_2_ with and without PDADMAC adsorption at different pH values was examined using Zetasizer Nano ZS (Malvern, Worcestershire, UK) in a background electrolyte of 1 mM KCl. 

The zeta (ζ) potential was calculated from electrophoretic mobility with Smoluchowski’s equation [44]

(1) $$\zeta =\frac{u_{e}\eta }{\varepsilon_{rs}\varepsilon_{0}}$$

where ζ is the ζ potential (mV), *u_e_* is the electrophoretic mobility (µm cm/sV), *η* is the dynamic viscosity of the liquid (mPa·s), *ε_rs_* is the relative permittivity constant of the electrolyte solution, and *ε*_0_ is the electric permittivity of the vacuum (8.854 × 10^−12^ F/m).

### 2.4. Adsorption Studies

All adsorption experiments were carried out by batch technique in 15 mL Falcon tubes at 25 ± 2 °C controlled by an air conditioner. Initial concentration of PDADMAC (20%) was precisely diluted with ultrapure water to a stock solution of 20 g/L (20,000 mg/L). Then, the stock solution was appropriately diluted according to experimental requirements. 

A 10 g/L SiO_2_ solution was fixed and thoroughly mixed in 10 mL of PDADMAC in the range of 0.200 to 12 g/L for 2 h. After that, the PDADMAC solution was separated by centrifugation at 6000 rpm for 20 min. The effects of pH and ionic strength on PDADMAC adsorption were systematically studied. The concentration of PDADMAC was quantitatively determined by a total of nitrogen measurement with photoluminescence technique using a TNM-1 (TOC-V_CPH_, Shimadzu, Kyoto, Japan). The adsorption capacity Γ (mg/g) of PDADMAC onto nanosilica was calculated by Equation (2)

(2) $$\Gamma =\frac{c_{i}-c_{e}}{m}\times 1000$$

where *C_i_* (g/L) and *C_e_ (*g/L) are the equilibrium concentration and final concentration of PDADMAC, respectively, and *m* (g/L) is adsorbent dosage. The experimental adsorption studies were carried out in triplicate. 

For adsorptive removal of AMX, a different adsorbent dosage was shaken well with 10 mL PDADMAC under optimum conditions to modify the silica surface. After that, the adsorbent was washed with pure water before AMX in different concentrations was added. The concentrations of AMX were determined by UV–vis spectroscopy with a quartz cuvette with a 1 cm optical path length using a spectrophotometer (UV-1650 PC, Shimadzu, Kyoto, Japan). The relationship between the absorbance and concentrations of AMX at a wavelength of 228 nm as standard calibration curves in different conditions with a correlation coefficient of at least 0.999 was confirmed. 

The removal efficiency (%R) of AMX was calculated by Equation (3)

(3) $$Removal\;efficiency\;(\%R)\;=\;\frac{C_{i}-C_{e}}{C_{i}}\times 100\%$$

The obtained isotherms were fitted by a general isotherm equation that could be applied to describe PDADMAC adsorption isotherms onto SiO_2_. The general isotherm equation is

(4) $$\Gamma =\frac{\Gamma_{\infty }k_{1}C(\frac{1}{n}+k_{2}C_{e}^{n-1})}{1+k_{1}C(1+k_{2}C_{e}^{n-1})}$$

where Γ (mg/g) is the adsorbed amount of PDADMAC, Γ_∞_ (mg/g) is the maximum adsorption, Γ_∞_ can be determined from the data of adsorption isotherm at high PDADMAC concentrations, *k*_1_ (g/mg) and *k*_2_ (g/mg)*^n^*^−1^ are equilibrium constants for first layer of adsorption and clusters of *n* molecules, and *C_e_* (g/L) denotes the equilibrium concentrations of PDADMAC in the aqueous solution. We used a trial-and-error method with Origin Professional 8 Edition software to find appropriate values of *k*_1_, *k*_2_, and *n* for every isotherm.

## 3. Results and Discussion

### 3.1. Characterizations of Nanosilica from Rice Husk

The nanosilica from rice husk was characterized by XRD, FTIR, and SEM.

**Figure 3:**
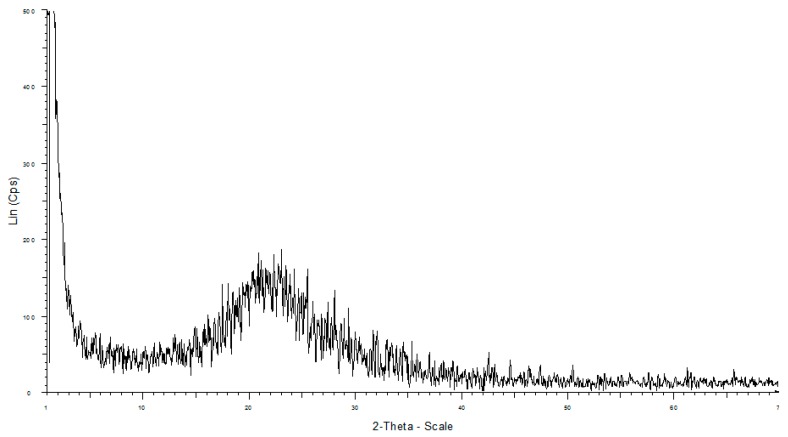
XRD pattern of nanosilica synthesized from rice husk.

The XRD pattern of the nanosilica particles fabricated from rice husk is shown in Figure 3. 

The powder diffraction pattern of synthesized silica indicates a broadened peak at 2θ = 20°–24°, which reveals the amorphous nature of the nanosilica particles [32].

**Figure 4:**
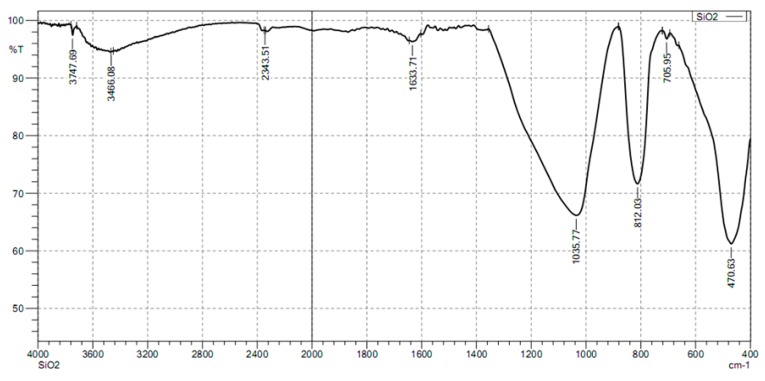
FTIR spectrum of nanosilica particles in the wave number range 400–4000 cm^−1^.

The FTIR spectrum of the nanosilica particles shown in Figure 4 indicates that the peaks at 1037.77, 812.03, and 470.63 cm^−1^ were due to the asymmetric, symmetric, and bending modes of SiO_2_, respectively [45]. The band at 3466.08 cm^−1^ and the peak at 1633.71 cm^−1^ for the sample are due to the –OH groups. These results confirm the existence of Si–O–Si stretch [32,45]. In addition, the FTIR spectrum of nanosilica revealed that unexpected peaks of any organic and inorganic compounds did not appear. In other words, the presence of silica is evident. 

**Figure 5:**
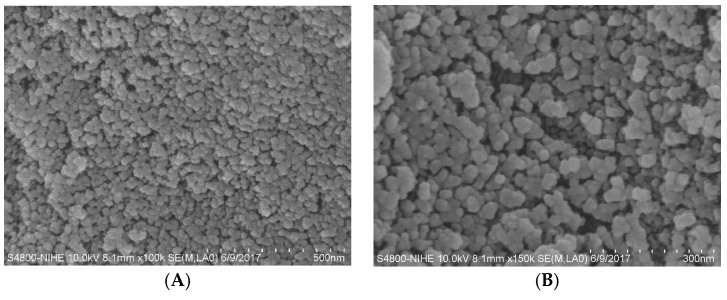
SEM images of SiO_2_ nanoparticles at different scales: (**A**) 500 nm; and (**B**) 300 nm.

The SEM images of silica in Figure 5 show that silica has nearly spherical particles. Based on SEM images, we can calculate an average diameter of silica particles in the range of 40–60 nm, demonstrating that silica synthesized from rice husk is a nanosized powder.

Based on the results of XRD, FTIR, and SEM, we demonstrate that silica nanoparticles are successfully synthesized from milled rice husk with a simple procedure. 

### 3.2. Adsorption of PDADMAC onto Nanosilica

#### 3.2.1. Effect of pH

**Figure 6:**
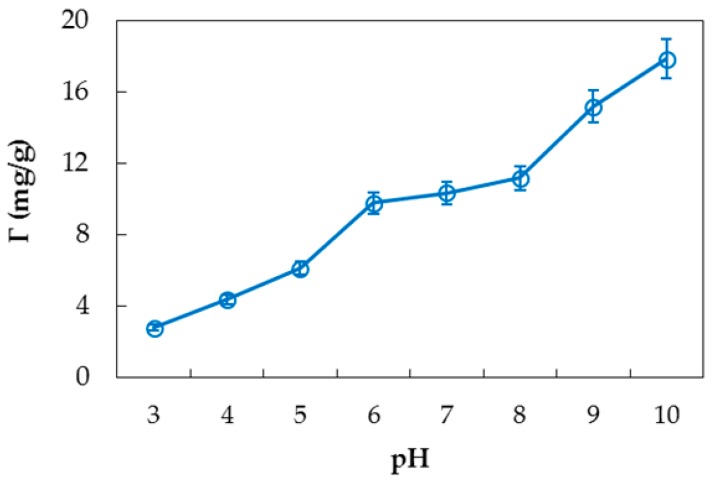
Effect of pH on PDADMAC adsorption on nanosilica. (*C_i_* (PDADMAC) = 1.0 g/L, adsorbent dosage 10 mg/mL, 100 mM KCl). Error bars show standard deviations of three replicates.

Figure 6 shows that adsorption of PDADMAC increases with an increase of pH from 3 to 10. With increasing pH, the surface of nanosilica has a higher negative charge that can enhance adsorption of strong polycation PDADMAC [46]. Further increased pH can cause dissolution of silica so that the standard deviations of replicates are high at higher pH. However, adsorption capacity at pH < 6 is much lower than that at high pH. Thus, a pH less than 10 and greater than 6 is better for the study of PDADMAC adsorption on nanosilica.

#### 3.2.2. Effect of Ionic Strength

Ionic strength strongly affects adsorption of PDADMAC, because adsorption of polyelectrolyte can be induced by both electrostatic and nonelectrostatic interactions [10]. The experimental PDADMAC adsorption on nanosilica was conducted in KCl from 0.1 mM to 100 mM at pH 6 and pH 10. 

**Figure 7:**
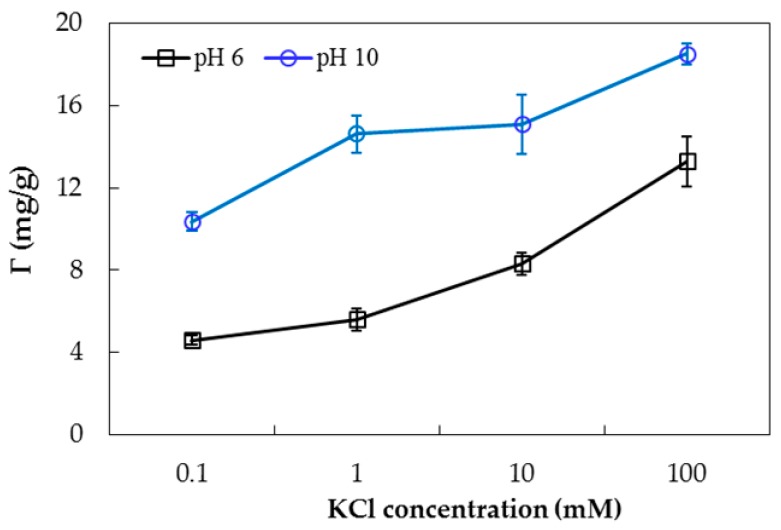
Effect of KCl concentration on PDADMAC adsorption on nanosilica (*C*_i_ (PDADMAC) = 1.0 g/L M, adsorbent dosage 10 mg/mL). Error bars show standard deviations of three replicates.

Figure 7 shows that the adsorption capacity of PDADMAC onto nanosilica increases with increasing KCl concentration at both pH 6 and pH 10. It suggests that both electrostatic and nonelectrostatic attraction can induce adsorption of PDADMAC on nanosilica. When salt is increased, the electrostatic attraction is screened, but polyelectrolyte with more loops and tails can form [10]. In order to evaluate the effect of ionic strength, adsorption isotherms were conducted, and this is discussed below. 

#### 3.2.3. Adsorption Isotherms

**Figure 8:**
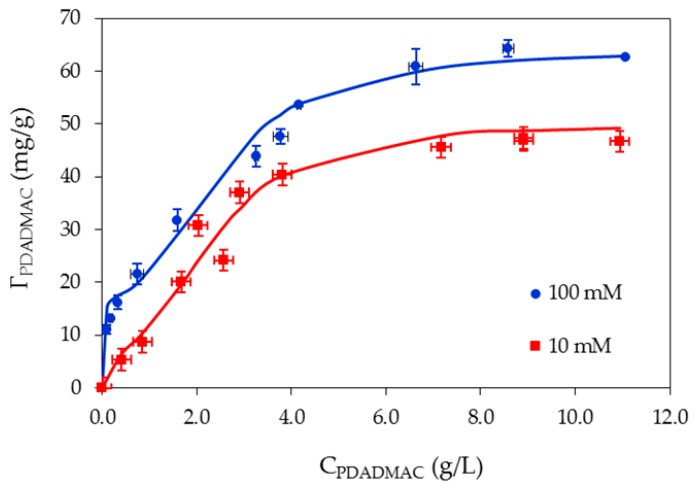
Adsorption isotherms of PDADMAC onto nanosilica at different KCl concentrations (pH 10). Points are experimental data; solid lines are the results of the two-step adsorption model. Error bars show standard deviations of three replicates.

The effect of ionic strength on adsorption of PDADMAC onto nanosilica is clearly demonstrated on the isotherms (Figure 8). At pH 10, adsorption of PDADMAC increases with increasing ionic strength. The adsorption capacity of PDADMAC at 100 mM KCl is always higher than that at 10 mM KCl.

For PDADMAC adsorption, an increase in salt concentration increases the number of cations K^+^ (counter ions) on the negatively-charged nanosilica surface, reducing the electrostatic effect of nanosilica and polycation (at pH 10). The electrostatic attraction between the positive charge of PDADMA^+^ ions and negatively-charged nanosilica surface is effectively screened by increasing salt concentration [47]. However, other interactions—such as hydrophobic, hydrogen bonding, surface complexation, and Van der Waals interactions—can induce adsorption [48]. This implies that adsorption of PDADMAC on nanosilica is controlled by both electrostatic and nonelectrostatic interactions, similar to adsorption of negatively charged polystyrene sulfonate onto a positively-charged alumina surface [10]. Polyelectrolyte adsorption in the presence of salt also releases counter ions to the bulk solution, so the driving force for adsorption can be induced by an increase of entropy [35,49].

Figure 8 shows that at different KCl concentrations, the experimental results of PDADMAC onto nanosilica can be represented well by general isotherm Equation (2) with the fitting parameters in Table 1 (solid lines in Figure 8). As shown in Table 1, increasing the ionic strength from 10 mM to 100 mM causes an increase in *k*_1_ (about 25 times), which shows that the number of sites increases for PDADMAC with increasing KCl concentration. Nevertheless, the value *k*_2_ does not change and is much lower than *k*_1_, suggesting that the amount of adsorbed loops and tails of polymer increases with an increase of salt. Furthermore, the value of n does not change significantly for PDADMAC adsorption. Our results are different from the case of polyelectrolyte adsorption onto cotton fiber, in which adsorption took place into a porous structure with a high value of *k*_2_. In our case, PDADMAC adsorption only occurred on the silica surface with a multilayer formation.

Adsorption of PDADMAC onto nanosilica at different ionic strengths reaches a plateau when the initial PDADMAC concentration is 10 g/L (1.0%). Therefore, PDADMAC at a concentration of 1.0% is used to modify the nanosilica surface for removal of antibiotic in the next section.

#### 3.2.4. Adsorption Mechanisms

Adsorption of polycation PDADMAC onto nanosilica from rice husk is discussed in detail based on the change in surface charge and functional groups by ζ potential and FTIR, respectively, as well as the adsorption isotherm (Section 3.2.3).

**Figure 9:**
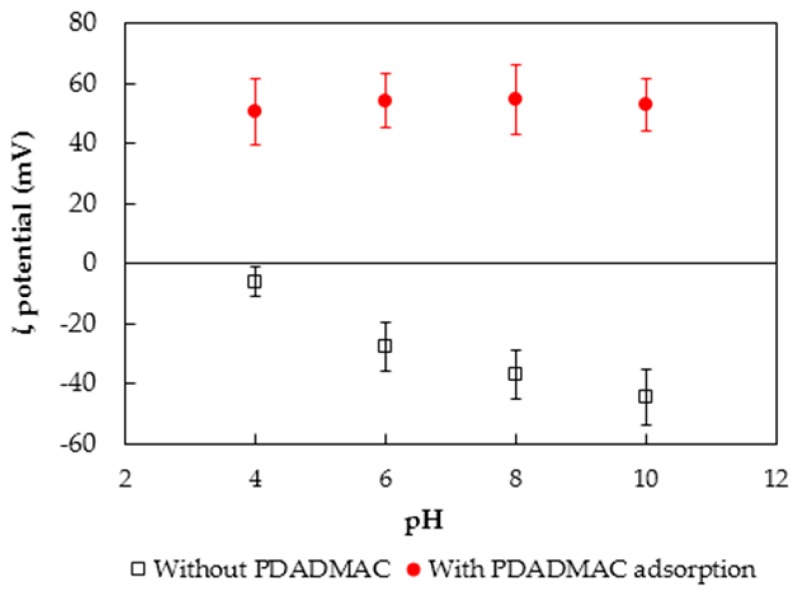
The ζ potentials of nanosilica particles without and with PDADMAC adsorption as a function of pH in 1 mM KCl background electrolyte.

Zeta (ζ) potential measurement calculated from electrophoretic mobility is useful for evaluating the charging behavior of many nano- and micro-sized materials [50,51]. The ζ potentials of silica nanoparticles without and with PDADMAC adsorption as a function of pH are indicated in Figure 9. Square points in Figure 9 show that the *ζ* potentials of nanosilica are negative and decrease with increasing pH, demonstrating that the surface charge of silica nanoparticles is negative. These results are in good agreement with the literature [43,52,53]. On the other hand, the ζ potentials of nanosilica after PDADMAC adsorption (circles in Figure 9) increase dramatically in the range of pH 4 to pH 10. Ζeta potential values of silica nanoparticles increase due to the presence of strong polycation ammonium groups. Due to the various cationic ammonium groups in the polyelectrolyte molecules, charge reversal takes place so that the charge of nanosilica is highly positive. The results of *ζ* potentials also indicate that PDADMAC molecules adsorbed onto the silica surface through electrostatic attraction. The charge of silica after PDADMAC adsorption is independent of pH, and polycation molecules strongly remain after centrifuging, which is good for adsorptive removal of anionic pollutants.

**Figure 10:**
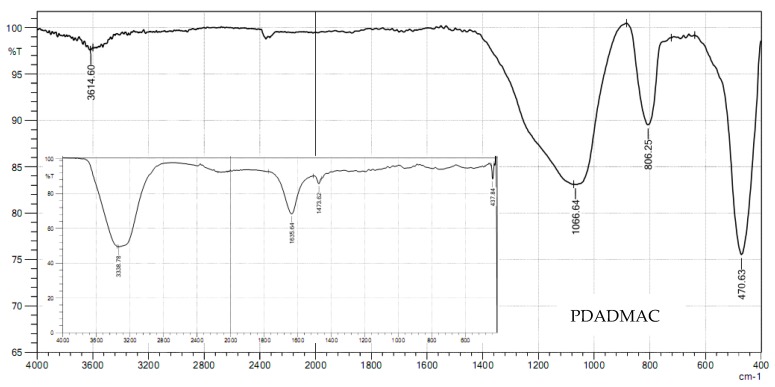
FTIR spectrum of nanosilica particles after PDADMAC adsorption in the wave number range of 400–4000 cm^–1^. The inset is an FTIR spectrum of PDADMAC solution.

The PDADMAC adsorption mechanism is also supported by the FTIR spectrum of nanosilica after adsorption (Figure 10). The peaks of the functional group N–H of ammonium ions in PDADMAC molecules at 1473.62 cm^−1^ (Figure 10, inset spectrum) disappeared after adsorption onto nanosilica [54]. Furthermore, the peaks of 812.03 cm^−1^ assigned for symmetric Si–O (Figure 4) shifted to a shorter wavelength (806.25 cm^–1^), while bending modes at 470.63 cm^–1^ did not change after PDADMAC adsorption. The results demonstrate that PDADMAC molecules attached to the SiO_2_ surface through electrostatic attraction and hydrophobic interaction.

**Figure 11:**
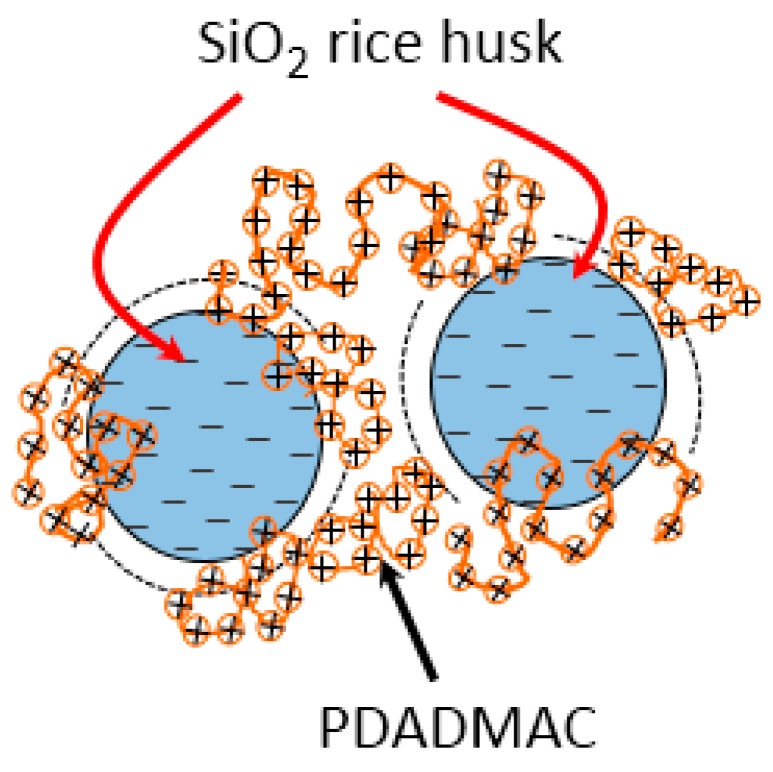
Cartoon representation of PDADMAC adsorption onto nanosilica rice husk.

Figure 11 shows a cartoon representation of the adsorbed structure of PDADAMC onto nanosilica, where the PDADMAC adsorbed onto silica via electrostatic attraction between positive PDADMAC molecules and the negatively charged SiO_2_ surface. This is similar to ammonium ion adsorption on graphite oxide/aluminum and graphite oxide/zirconium-aluminum polyoxycation composites, where the interaction is via hydrogen bonding with surface structure oxygen and functional groups [55]. In addition, PDADMAC molecules can create a lateral interactions and bridge the silica particles due to the interactions of hydrocarbon chains. Thus, PDADMAC adsorption onto nanosilica is induced by both electrostatic and non-electrostatic interactions.

### 3.3. Adsorptive Removal of Amoxicillin Using PDADMAC-Modified Nanosilica

#### 3.3.1. Influence of Contact Time

**Figure 12:**
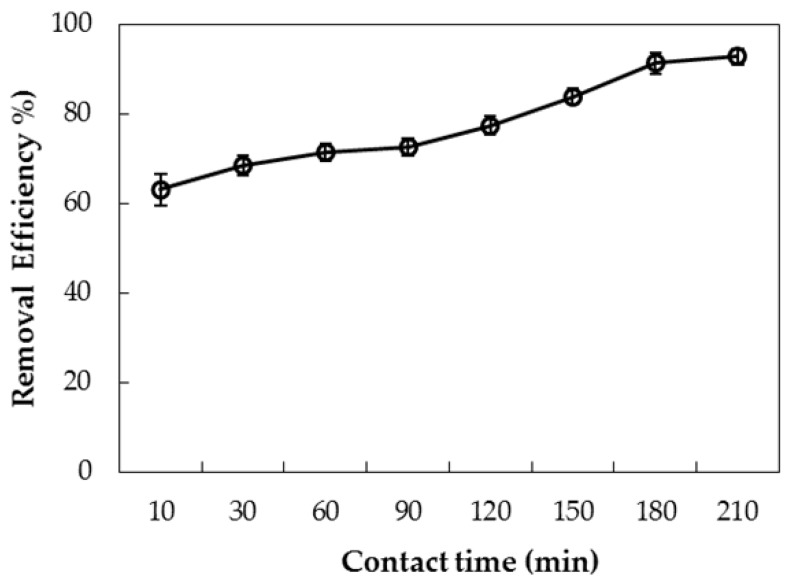
Influence of contact time on removal of amoxicillin (AMX) using PDADMAC-modified silica (PMS). Error bars show standard deviations of three replicates.

Contact time strongly induces to equilibrium of the adsorption process. The effect of contact time on the adsorptive removal of AMX using PDADMAC-modified nanosilica (PMS) is presented in Figure 12. As can be seen, removal of AMX increases with an increase of the contact time from 10 min to 180 min. After 180 min, removal changes insignificantly. This suggests that the adsorption reaches equilibrium at 180 min. Thus, 180 min is chosen for removal of AMX using PMS.

#### 3.3.2. Influence of pH 

**Figure 13:**
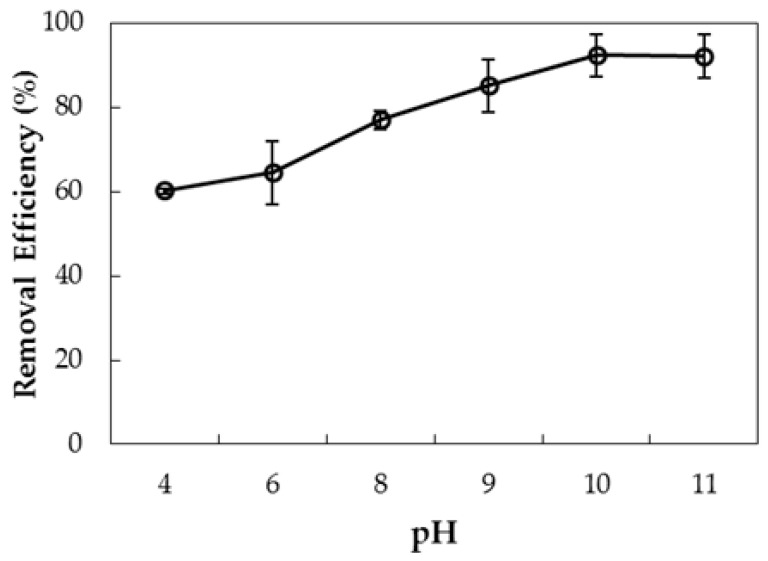
Influence of pH on removal of AMX using PMS. Error bars show standard deviations of three replicates.

The pH of the solution plays an important role in adsorption of AMX onto PMS, because it can affect the PMS surface charge and the AMX charging behavior. The effect of initial pH on removal of AMX using PMS was investigated in the range of pH 4–11 in 1 mM KCl (Figure 13). 

As seen in Figure 13, removal of AMX increases with increasing pH of the solution from 4 to 10, then slightly decreases. At lower pH, the form of AMX has a neutral charge from pH 2.7 to 7.5; at pH higher than 7.5, AMX has a negative charge [13] that can easily adsorb onto the positively-charged PMS. However, dissolution of silica can take place at pH > 10, so removal decreases. Thus, optimum pH for removal of AMX by PMS is pH 10.

#### 3.3.3. Influence of Adsorbent Dosage

The adsorbent dosage has a significant effect on the adsorption process, because it affects the total surface area of the adsorbent and the number of binding sites. The amount of PMS varied from 2.5 to 50 mg/mL.

**Figure 14:**
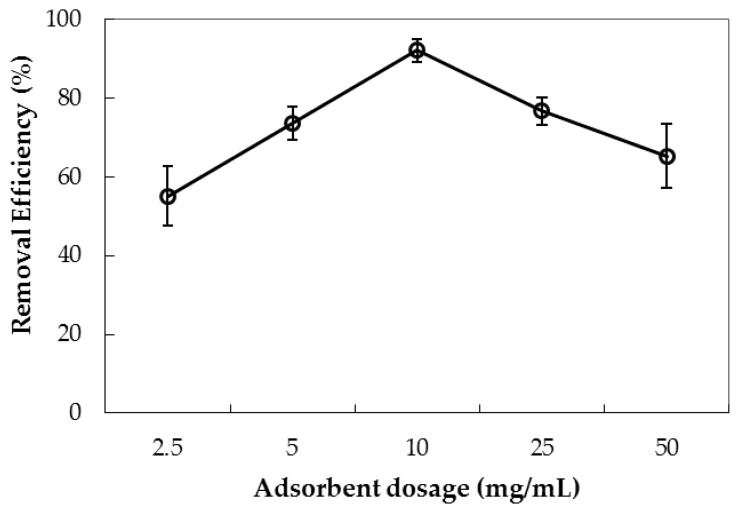
Influence of adsorbent dosage on removal of AMX using PMS. Error bars show standard deviations of three replicates.

Figure 14 indicates that removal of AMX using PMS increases rapidly with increasing adsorbent dosage from 2.5 to 10 mg/mL. This may be explained by a large number of available binding sites for adsorption or an increased net specific surface area with increased dosage [56]. However, further increasing the adsorbent dosage over 10 mg/mL results in significantly decreased removal efficiency due to the very fast aggregation of silica particles at high adsorbent dosage [43,53]. The error bar showing the deviations of triplicates with 10 mg/mL is smaller than those of other dosages. Thus, optimum adsorbent dosage is found to be 10 mg/mL, and it is fixed for adsorptive removal studies.

### 3.4. Adsorptive Removal of AMX onto Silica without and with Surface Modification with PDADMAC 

**Figure 15:**
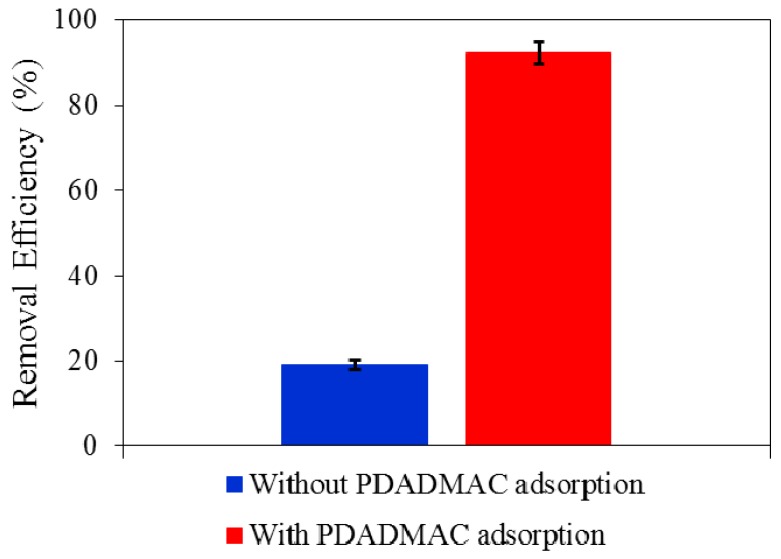
Removal of AMX using silica without and with PDADMAC modification. Error bars show standard deviations of three replicates.

According to the results of PDADMAC adsorption on nanosilica (Section 3.2), the conditions to modify silica surface are 1% PDADMAC, pH 10, and 100 mM KCl. As a result, the surface charge of nanosilica is positive due to the presence of the polycation. Figure 15 shows that removal of AMX (at an initial concentration of 10 mg/L) in 1 mM KCl increases significantly from 19.1% to 92.3% after surface modification of nanosilica by PDADMAC. AMX adsorption onto bare nanosilica is highest at pH 11, at which low adsorption can be promoted by lateral interaction with the negatively-charged AMX. AMX adsorption onto PMS was significantly enhanced by electrostatic attraction between the negatively charged AMX and the positively charged PMS surface. This implies that PDADMAC-modified nanosilica rice husk can be used as a potential adsorbent more than bare nanosilica. 

**Figure 16:**
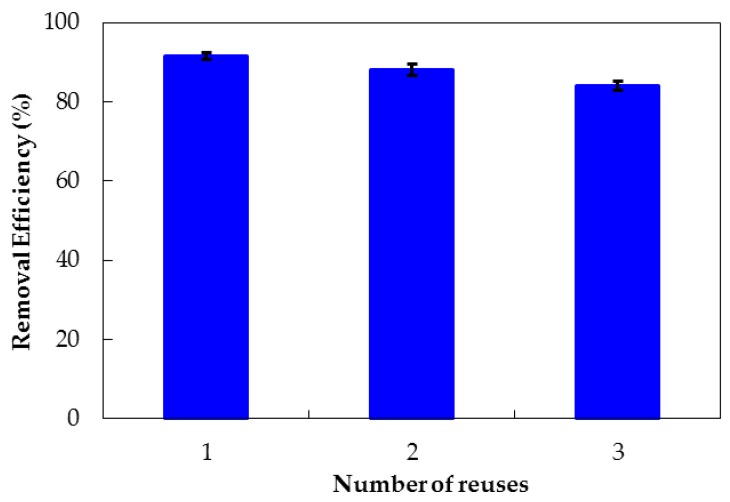
Efficiency of removing AMX with PMS after being reused three times. Error bars show standard deviations of three replicates of independently-prepared adsorbent.

Although the silica synthesized from rice husk is a cheap adsorbent, the regeneration experiment is important to evaluate the stability and reuse potential of PMS. Thus, three sets of experiments were performed. The number of reuses in our case is quite small compared with L-phenylalanine methyl ester-imprinted polymer adsorption regeneration cycles [57], or 10 cycles of Pb^2+^ on alginate halloysite nanotube nanocomposite beads [58], but it is important to evaluate the economic and the environmental aspects of PMS for antibiotic removal. The AMX loaded onto PMS was released in 0.1 M KOH before being washed with ultrapure water for subsequent AMX adsorption experiments with the same conditions. Figure 16 shows that the efficiency of removing AMX with PMS after being reused three times decreased slightly. Removal efficiency was still greater than 83%. The decrease of removal efficiency is similar to the adsorption/desorption of arsenic on the material magnetic silica [59]. However, the removal efficiency is suitable for reuse PMS. This implies that PDADMAC is irreversibly attached to the nanosilica rice husk. In other words, PMS is a novel adsorbent in terms of economic and environmental aspects. 

### 3.5. Comparison of the Effectiveness of PMS and Other Adsorbents

Some published articles have reported on removal of AMX using different kinds of adsorbents. However, adsorptive removal of AMX using PMS has not been studied. In addition, the PMS used in the present study has very high removal efficiency compared to adsorbents (Table 2). PMS is also a cheap adsorbent because rice husk is an agricultural byproduct. This demonstrates that PMS is a novel material for the removal of antibiotics from aqueous solutions.

## 4. Conclusions

In the present study, we investigated adsorption of the polyelectrolyte polydiallyldimethylammonium chloride (PDADMAC) onto nanosilica (SiO_2_). Nanosilica that was successfully fabricated from rice husk was characterized by X-ray diffraction (XRD), Fourier-transform infrared spectroscopy (FTIR), and scanning electron microscopy (SEM). Adsorption of PDADMAC onto SiO_2_ increased with increasing pH due to the less negative surface charge of SiO_2_ at lower pH, while PDADMAC adsorption onto SiO_2_ decreased with decreasing KCl concentration from 0.1 to 100 mM. Experimental results of adsorption isotherms of PDADMAC onto silica at different ionic strengths were well represented by a two-step adsorption model. Based on adsorption isotherms, surface charge effects by evaluating the change in ζ potentials, and the surface modification by FTIR, we suggest that the adsorption mechanism of PDADMAC onto SiO_2_ is induced by electrostatic attraction between cationic polymer and the negatively charged SiO_2_ surface and non-electrostatic interactions, including hydrophobic and lateral interactions. Furthermore, surface modification of SiO_2_ by pre-adsorption of PDADMAC caused a significant increase in efficiency of removing amoxicillin (AMX). The optimum conditions of contact time, adsorbent dosage, and pH for removing AMX using PDADMAC-modified SiO_2_ were found to be 180 min, pH 10, and 10 mg/mL, respectively. Under optimum adsorption conditions, AMX removal from aqueous solution increased significantly from 19.1% to 92.3%. Our results demonstrate that PDADMAC-modified nanosilica synthesized from rice husk is a novel adsorbent for the removal of beta lactam antibiotics from aqueous solutions.

## Figures and Tables

**Table 1:** Fit parameters for polydiallyldimethylammonium chloride (PDADMAC) adsorption onto nanosilica

CKCl (mM)	Γ_∞_ (mg/g)	*k*_1_ (g/mg)	*k*_2_ (g/mg) *^n^*^−1^	*n*
10	50	2.1	0.1	3.6
100	64	50	0.1	3.5

**Table 2:** Adsorption capacity and removal efficiency of polydiallyldimethylammonium chloride-modified silica (PMS) and other absorbents for removing amoxicillin (AMX).

Adsorbent	Adsorption Capacity (mg/g)	Removal Efficiency (%)	References
Activated carbon	7.367	50.26	[60]
Chitosan beads	8.71	NF	[61]
Organobentonite	26.18	61.5	[13]
Bentonite	18.75	88.01	[31]
Organoclays	24.29	34.80	[62]
PMS	7.50	92.30	This study

NF: no information.

## References

[1] Ayodele O.B. (2013). Effect of phosphoric acid treatment on kaolinite supported ferrioxalate catalyst for the degradation of amoxicillin in batch photo-Fenton process. Appl. Clay Sci..

[2] Fakhri A., Adami S. (2014). Adsorption and thermodynamic study of Cephalosporins antibiotics from aqueous solution onto MgO nanoparticles. J. Taiwan Inst. Chem. Eng..

[3] Guo R., Chen J. (2015). Application of alga-activated sludge combined system (AASCS) as a novel treatment to remove cephalosporins. Chem. Eng. J..

[4] Michael I., Rizzo L., McArdell C.S., Manaia C.M., Merlin C., Schwartz T., Dagot C., Fatta-Kassinos D. (2013). Urban wastewater treatment plants as hotspots for the release of antibiotics in the environment: A review. Water Res..

[5] Akmehmet Balcıoğlu I., Ötker M. (2003). Treatment of pharmaceutical wastewater containing antibiotics by O3 and O3/H2O2 processes. Chemosphere.

[6] Herrmann J.M., Guillard C., Pichat P. (1993). Heterogeneous photocatalysis: An emerging technology for water treatment. Catal. Today.

[7] Santiago-Morales J., Gómez M.J., Herrera-López S., Fernández-Alba A.R., García-Calvo E., Rosal R. (2013). Energy efficiency for the removal of non-polar pollutants during ultraviolet irradiation, visible light photocatalysis and ozonation of a wastewater effluent. Water Res..

[8] Sousa V.M., Manaia C.M., Mendes A., Nunes O.C. (2013). Photoinactivation of various antibiotic resistant strains of Escherichia coli using a paint coat. J. Photochem. Photobiol. Chem..

[9] Dutta M., Dutta N.N., Bhattacharya K.G. (1999). Aqueous phase adsorption of certain beta-lactam antibiotics onto polymeric resins and activated carbon. Sep. Purif. Technol..

[10] Pham T.D., Kobayashi M., Adachi Y. (2014). Adsorption of Polyanion onto Large Alpha Alumina Beads with Variably Charged Surface. Adv. Phys. Chem..

[11] Pham T.D., Kobayashi M., Adachi Y. (2015). Adsorption of anionic surfactant sodium dodecyl sulfate onto alpha alumina with small surface area. Colloid Polym. Sci..

[12] Pham T.D., Kobayashi M., Adachi Y. (2015). Adsorption characteristics of anionic azo dye onto large α-alumina beads. Colloid Polym. Sci..

[13] Zha S.X., Zhou Y., Jin X., Chen Z. (2013). The removal of amoxicillin from wastewater using organobentonite. J. Environ. Manag..

[14] Gwenzi W., Chaukura N., Noubactep C., Mukome F.N.D. (2017). Biochar-based water treatment systems as a potential low-cost and sustainable technology for clean water provision. J. Environ. Manag..

[15] Ribau Teixeira M., Camacho F.P., Sousa V.S., Bergamasco R. (2017). Green technologies for cyanobacteria and natural organic matter water treatment using natural based products. J. Clean. Prod..

[16] Razali M., Kim J.F., Attfield M., Budd P.M., Drioli E., Lee Y.M., Szekely G. (2015). Sustainable wastewater treatment and recycling in membrane manufacturing. Green Chem..

[17] Figueroa R.A., MacKay A.A. (2005). Sorption of Oxytetracycline to Iron Oxides and Iron Oxide-Rich Soils. Environ. Sci. Technol..

[18] Mon J., Flury M., Harsh J.B. (2006). Sorption of four triarylmethane dyes in a sandy soil determined by batch and column experiments. Geoderma.

[19] Wang S., Wang H. (2015). Adsorption behavior of antibiotic in soil environment: A critical review. Front. Environ. Sci. Eng..

[20] Pham T.D., Do T.T., Ha V.L., Doan T.H.Y., Nguyen T.A.H., Mai T.D., Kobayashi M., Adachi Y. (2017). Adsorptive removal of ammonium ion from aqueous solution using surfactant-modified alumina. Environ. Chem..

[21] Adak A., Pal A., Bandyopadhyay M. (2005). Spectrophotometric determination of anionic surfactants in wastewater using acridine orange. Ind. J. Chem. Technol..

[22] Aloulou F., Boufi S., Beneventi D. (2004). Adsorption of organic compounds onto polyelectrolyte immobilized-surfactant aggregates on cellulosic fibers. J. Colloid Interface Sci..

[23] Mishael Y.G., Dubin P.L. (2005). Uptake of Organic Pollutants by Silica−Polycation-Immobilized Micelles for Groundwater Remediation. Environ. Sci. Technol..

[24] Mishael Y.G., Dubin P.L., De Vries R., Kayitmazer A.B. (2007). Effect of Pore Size on Adsorption of a Polyelectrolyte to Porous Glass. Langmuir.

[25] Adamczyk Z., Zembala M., Michna A. (2006). Polyelectrolyte adsorption layers studied by streaming potential and particle deposition. J. Colloid Interface Sci..

[26] Blokhus A.M., Djurhuus K. (2006). Adsorption of poly(styrene sulfonate) of different molecular weights on α-alumina: Effect of added sodium dodecyl sulfate. J. Colloid Interface Sci..

[27] Hoogeveen N.G., Stuart M.A.C., Fleer G.J. (1996). Polyelectrolyte Adsorption on Oxides: I. Kinetics and Adsorbed Amounts. J. Colloid Interface Sci.

[28] Ahmed M.B., Zhou J.L., Ngo H.H., Guo W. (2015). Adsorptive removal of antibiotics from water and wastewater: Progress and challenges. Sci. Total Environ..

[29] Homem V., Santos L. (2011). Degradation and removal methods of antibiotics from aqueous matrices—A review. J. Environ. Manag..

[30] Li B., Zhang T. (2010). Biodegradation and Adsorption of Antibiotics in the Activated Sludge Process. Environ. Sci. Technol..

[31] Putra E.K., Pranowo R., Sunarso J., Indraswati N., Ismadji S. (2009). Performance of activated carbon and bentonite for adsorption of amoxicillin from wastewater: Mechanisms, isotherms and kinetics. Water Res..

[32] Pham T.D., Vu C.M., Choi H.J. (2017). Enhanced fracture toughness and mechanical properties of epoxy resin with rice husk-based nano-silica. Polym. Sci. Ser. A.

[33] Wang Y., Banziger J., Dubin P.L., Filippelli G., Nuraje N. (2001). Adsorptive Partitioning of an Organic Compound onto Polyelectrolyte-Immobilized Micelles on Porous Glass and Sand. Environ. Sci. Technol..

[34] Zadaka D., Mishael Y.G., Polubesova T., Serban C., Nir S. (2007). Modified silicates and porous glass as adsorbents for removal of organic pollutants from water and comparison with activated carbons. Appl. Clay Sci..

[35] Guzman E., Ritacco H., Rubio J.E.F., Rubio R.G., Ortega F. (2009). Salt-induced changes in the growth of polyelectrolyte layers of poly(diallyl-dimethylammonium chloride) and poly(4-styrene sulfonate of sodium). Soft Matter.

[36] Langmuir I. (1918). The adsorption of gases on plane surfaces of glass, mica and platinum. J. Am. Chem. Soc..

[37] Freundlich H. (1907). Über die adsorption in Lösungen. Z. Phys. Chem..

[38] Barrett E.P., Joyner L.G., Halenda P.P. (1951). The Determination of Pore Volume and Area Distributions in Porous Substances. I. Computations from Nitrogen Isotherms. J. Am. Chem. Soc..

[39] Zhu B.-Y., Gu T. (1991). Surfactant adsorption at solid-liquid interfaces. Adv. Colloid Interface Sci..

[40] Hoffmann I., Oppel C., Gernert U., Barreleiro P., Von Rybinski W., Gradzielski M. (2012). Adsorption Isotherms of Cellulose-Based Polymers onto Cotton Fibers Determined by Means of a Direct Method of Fluorescence Spectroscopy. Langmuir.

[41] Ndong R., Russel W. (2012). Linear viscoelasticity of ZrO_2_ nanoparticle dispersions with associative polymers. Rheol. Acta.

[42] Guzmán E., Ritacco H.A., Ortega F., Rubio R.G. (2012). Growth of Polyelectrolyte Layers Formed by Poly(4-styrenesulfonate sodium salt) and Two Different Polycations: New Insights from Study of Adsorption Kinetics. J. Phys. Chem. C.

[43] Kobayashi M., Skarba M., Galletto P., Cakara D., Borkovec M. (2005). Effects of heat treatment on the aggregation and charging of Stöber-type silica. J. Colloid Interface Sci..

[44] Delgado A.V., González-Caballero F., Hunter R.J., Koopal L.K., Lyklema J. (2007). Measurement and interpretation of electrokinetic phenomena. J. Colloid Interface Sci..

[45] Kannan C., Sundaram T., Palvannan T. (2008). Environmentally stable adsorbent of tetrahedral silica and non-tetrahedral alumina for removal and recovery of malachite green dye from aqueous solution. J. Hazard. Mater..

[46] Čakara D., Kobayashi M., Skarba M., Borkovec M. (2009). Protonation of silica particles in the presence of a strong cationic polyelectrolyte. Colloids Surf. A Physicochem. Eng. Asp..

[47] Matsumoto T., Adachi Y. (1998). Effect of Ionic Strength on the Initial Dynamics of Flocculation of Polystyrene Latex with Polyelectrolyte. J. Colloid Interface Sci..

[48] Mészáros R., Thompson L., Bos M., De Groot P. (2002). Adsorption and Electrokinetic Properties of Polyethylenimine on Silica Surfaces. Langmuir.

[49] Guzmán E., Cavallo J.A., Chuliá-Jordán R., Gómez C., Strumia M.C., Ortega F., Rubio R.G. (2011). pH-Induced Changes in the Fabrication of Multilayers of Poly(acrylic acid) and Chitosan: Fabrication, Properties, and Tests as a Drug Storage and Delivery System. Langmuir.

[50] Kobayashi M. (2008). Electrophoretic mobility of latex spheres in the presence of divalent ions: experiments and modeling. Colloid Polym. Sci..

[51] Yamaguchi A., Kobayashi M. (2016). Quantitative evaluation of shift of slipping plane and counterion binding to lysozyme by electrophoresis method. Colloid Polym. Sci..

[52] Huang Y., Yamaguchi A., Pham T.D., Kobayashi M. (2018). Charging and aggregation behavior of silica particles in the presence of lysozymes. Colloid Polym. Sci..

[53] Kobayashi M., Juillerat F., Galletto P., Bowen P., Borkovec M. (2005). Aggregation and Charging of Colloidal Silica Particles:  Effect of Particle Size. Langmuir.

[54] Kochameshki M.G., Marjani A., Mahmoudian M., Farhadi K. (2017). Grafting of diallyldimethylammonium chloride on graphene oxide by RAFT polymerization for modification of nanocomposite polysulfone membranes using in water treatment. Chem. Eng. J..

[55] Seredych M., Bandosz T.J. (2008). Adsorption of ammonia on graphite oxide/aluminium polycation and graphite oxide/zirconium–aluminium polyoxycation composites. J. Colloid Interface Sci..

[56] Mazloomi F., Jalali M. (2016). Ammonium removal from aqueous solutions by natural Iranian zeolite in the presence of organic acids, cations and anions. J. Environ. Chem. Eng..

[57] Kupai J., Razali M., Buyuktiryaki S., Kecili R., Szekely G. (2017). Long-term stability and reusability of molecularly imprinted polymers. Polym. Chem..

[58] Chiew C.S.C., Yeoh H.K., Pasbakhsh P., Poh P.E., Tey B.T., Chan E.S. (2016). Stability and reusability of alginate-based adsorbents for repetitive lead (II) removal. Polym. Degrad. Stab..

[59] Saiz J., Bringas E., Ortiz I. (2014). New Functionalized Magnetic Materials for As5+ Removal: Adsorbent Regeneration and Reuse. Ind. Eng. Chem. Res..

[60] Pouretedal H.R., Sadegh N. (2014). Effective removal of Amoxicillin, Cephalexin, Tetracycline and Penicillin G from aqueous solutions using activated carbon nanoparticles prepared from vine wood. J. Water Process Eng..

[61] Adriano W.S., Veredas V., Santana C.C., Gonçalves L.R.B. (2005). Adsorption of amoxicillin on chitosan beads: Kinetics, equilibrium and validation of finite bath models. Biochem. Eng. J..

[62] Jin X., Zha S., Li S., Chen Z. (2014). Simultaneous removal of mixed contaminants by organoclays—Amoxicillin and Cu(II) from aqueous solution. Appl. Clay Sci..

